# Disease Control of Delay SEIR Model with Nonlinear Incidence Rate and Vertical Transmission

**DOI:** 10.1155/2013/830237

**Published:** 2013-12-12

**Authors:** Yan Cheng, Qiuhui Pan, Mingfeng He

**Affiliations:** ^1^School of Mathematical Sciences, Dalian University of Technology, Dalian 116024, China; ^2^Department of Mathematics, Tonghua Normal University, Tonghua 136000, China; ^3^School of Innovation Experiment, Dalian University of Technology, Dalian 116024, China

## Abstract

The aim of this paper is to develop two delayed SEIR epidemic models with nonlinear incidence rate, continuous treatment, and impulsive vaccination for a class of epidemic with latent period and vertical transition. For continuous treatment, we obtain a basic reproductive number *ℜ*_0_ and prove the global stability by using the Lyapunov functional method. We obtain two thresholds ℜ* and *ℜ*_∗_ for impulsive vaccination and prove that if ℜ* < 1, then the disease-free periodic solution is globally attractive and if *ℜ*_∗_ > 1, then the disease is permanent by using the comparison theorem of impulsive differential equation. Numerical simulations indicate that pulse vaccination strategy or a longer latent period will make the population size infected by a disease decrease.

## 1. Introduction 

Mathematical models describing the population dynamics of infectious diseases have been playing an important role in understanding epidemiological patterns and disease control. Researchers have studied the epidemic models by ordinary differential equations [1–3] and the references cited therein. A customarily epidemic model is susceptible, infectious, and recovered model (SIR for short) [4–7]. But in real life, many diseases have a period of incubation time inside the hosts before the hosts become infectious; if we include incubation period of the hosts, the model is described as SEIR model. As tuberculosis (TB), measles and so on, a susceptible individual becomes exposed (infected but not infective) by adequate contact with an infectious individual. SEIR infections disease model has been studied by many authors for its important biological meaning [8–13]. In [13], the authors considered the following delayed SEIR epidemic model:

(1) $$\begin{array}{c} S^{\prime }\left(t\right)=\Lambda -\mu S\left(t\right)-\frac{\beta S\left(t\right)I\left(t\right)}{1+\alpha I\left(t\right)}+\delta R\left(t\right),\\ E^{\prime }\left(t\right)=\frac{\beta S\left(t\right)I\left(t\right)}{1+\alpha I\left(t\right)}-\frac{\beta e^{-\mu \tau }S\left(t-\tau \right)I\left(t-\tau \right)}{1+\alpha I\left(t-\tau \right)}-\mu E,\\ I^{\prime }\left(t\right)=\frac{\beta e^{-\mu \tau }S\left(t-\tau \right)I\left(t-\tau \right)}{1+\alpha I\left(t-\tau \right)}-\left(\mu +\gamma \right)I,\\ R^{\prime }\left(t\right)=\gamma I-\left(\mu +\delta \right)R\left(t\right), \end{array}$$

where *S*(*t*), *E*(*t*), *I*(*t*), and *R*(*t*) represent the number of individuals who are susceptible, exposed, infected, and removed, respectively. The parameters Λ, *β*, *γ*, and *μ* are positive constants, and here Λ is the constant recruitment rate into the population, *β* is the contact rate, *μ* is the birth and death rate, *γ* is the removal rate. *τ* > 0 represents a time delay describing the latent period of the disease and the term (*βe* ^−*μτ*^ *S*(*t* − *τ*)*I*(*t* − *τ*))/(1 + *αI*(*t* − *τ*)) represents the individuals surviving in the latent period *τ* and becoming infective at time *t*. The sufficient conditions are obtained for the global asymptotic stability of the endemic equilibrium.

In the study of epidemic model, the spread of an infectious disease is a crucial issue, which depends on both the population behavior and the infectivity of the disease. These two aspects are captured in the incidence rate of a disease. In many epidemiological models, the incidence rate is described as mass action incidence with bilinear interactions given by *βSI*, where *β* is the probability of transmission per contact and *S* and *I* represent the susceptible and infected populations, respectively. This contact law is more appropriate for a few of infected individuals; when the size of infected individuals is increasing, the underlying assumption of homogeneous mixing may not be valid. In fact, with the increase of infected populations, the susceptible individual will take measure to prevent unbounded contact rates. In [14], Anderson and May proposed a saturated incidence rate of the form *βSI*/(1 + *αS*) in which *βSI* measures the infection force of the disease and 1/(1 + *αS*) measures the inhibition effect from the behavioral change of the susceptible individuals. The same as the nonlinear incidence rates of the form *kI* ^*p*^ *S* ^*q*^ were investigated by Liu et al. [15, 16].

In real life, some diseases may be transferred through horizontal transmission and vertical transmission (disease is the passing of an infection to offspring of infected parents). The offspring of infected parents may already be infected with the disease at birth, so many infections in nature transmit through both horizontal and vertical modes, such as tuberculosis (TB), rubella, hepatitis B, and AIDS [17–21].

Vaccination and treatment are important strategy for the elimination of infectious diseases. Recently, pulse vaccination has been confirmed as an effective method to prevent the spread of the disease [22–24]. Theoretical results show that the pulse vaccination strategy can be distinguished from the conventional strategies in leading to disease eradication at relatively low values of vaccination [25]. The study of vaccination, treatment, and associated behavioral changes related to disease transmission has been the subject of intense theoretical analysis.

The literature on SEIR model with nonlinear incidence, constant infectious period, impulsive vaccination, dealing with the analysis of disease that is vertically and horizontally transmitted is not extensive [17, 19]. But, in fact, under the situation of disease with vertical transmission, the continuous treatment should be considered for the infected, and impulsive vaccination to the susceptible, newborns of the susceptible, exposed and the removed, and newborns of infected which not be vertical infected.

Motivated by the literature above, we introduce delay epidemic models with nonlinear incidence rates of the form *βS* ^*p*^ *I*, and we also considered the constant latency period and vertically and horizontally in (2). The purpose of this paper is to study the nonlinear dynamics of system, and we consider two different strategies to the model which are constant treatment and pulse vaccination to the newborns and susceptible,

(2) $$\begin{array}{c} S^{\prime }\left(t\right)=bm\left(S+R+E\right)-\beta S^{p}I-bS+q^{\prime }\delta I,\\ E^{\prime }\left(t\right)=\beta S^{p}I-\beta e^{-b\tau }S^{p}\left(t-\tau \right)I\left(t-\tau \right)-bE,\\ I^{\prime }\left(t\right)=\beta e^{-b\tau }S^{p}\left(t-\tau \right)I\left(t-\tau \right)+q\delta I-\delta I-\gamma I-\frac{d}{T}I,\\ R^{\prime }\left(t\right)=\gamma I-bR+bm^{\prime }\left(S+R+E\right)+\frac{d}{T}I. \end{array}$$

The basic assumptions are as follows.

- The total population size at time *t* (day) is denoted by *N* = *S* + *E* + *I* + *R*. For $\dot{N}=0$, this shows that the total population has a constant size. Without loss of generality, we assume in this paper *N* = 1. The newborns of *S*,   *E*, and *R* are susceptible individuals, and the newborns of *I* who are not vertically infected are also susceptible individuals.
- The positive constant *b* (per day) denotes the death rate and birth rate of susceptible, exposed, and recovered individuals. The positive constant *δ* (per day) denotes the death rate and birth rate of infective individuals. The positive constant *γ* (per day) is the natural recovery rate of infective individuals. The positive constant *q*  (*q* ≤ 1) (per day) is the vertical transmission rate, and note *q*′ = 1 − *q*, *q*′ < *q* (per day), and then 0 < *q*′ < 1. Fraction *m*′ of all newborns with mothers in the susceptible, exposed, and recovered classes are vaccinated and appeared in the recovered class, while the remaining fraction, *m* = 1 − *m*′, appears in the susceptible class; suppose *bm* > *q*′*δ*.  (*d*/*T*) is the proportion of those cured successfully.
- The incidence rate is described by a nonlinear function *βS* ^*p*^ *I* where *β* (per day) is a positive constant describing the infection rate. *τ* > 0 is the length of the latent period and the term *βe* ^−*bτ*^ *S* ^*p*^(*t* − *τ*)*I*(*t* − *τ*) reflects the fact that an individual is surviving in the latent period *τ* and becoming infective at time *t*.

The remaining part of this paper is organized as follows. In Section 2, we investigate the global stability of the endemic equilibrium of (2) by using Rouches theorem and Lyapunov-LaSalle type theorem. The global asymptotic stability of disease-free periodic solution and the conditions for the permanence of the disease by comparison techniques are described in Section 3. Numerical simulations are presented in Section 4. In Section 5, we conclude this paper with some remarks.

## 2. Continuous Treatment Strategy of the SEIR Model 

In this section, we consider a continuous treatment of SEIR model with constant latent period and nonlinear incidence rate. By using *S* + *R* + *E* = 1 − *I*, notice that first and third equations of system (2) do not contain the variables *E* and *R*; therefore, system (2) is equivalent to the following 2-dimensional system:

(3) $$\begin{array}{c} S^{\prime }\left(t\right)=bm\left(1-I\right)-\beta S^{p}I-bS+q^{\prime }\delta I,\\ I^{\prime }\left(t\right)=\beta e^{-b\tau }S^{p}\left(t-\tau \right)I\left(t-\tau \right)+q\delta I-\delta I-\gamma I-\frac{d}{T}I. \end{array}$$

### 2.1. Disease-Free Equilibrium and Its Stability

First, we define

(4) $$\begin{array}{c} {ℜ}_{0}=\frac{\beta m^{p}e^{-b\tau }}{\delta -q\delta +\gamma +\left(d/T\right)}. \end{array}$$

**Theorem 1**

If *ℜ*_0_ < 1, the disease-free equilibrium *E*_0_(*m*, 0) of system (3) is locally asymptotically stable for all *τ* ≥ 0; if *ℜ*_0_ > 1, the disease-free equilibrium *E*_0_(*m*, 0) is unstable.

**Proof**

Steady states of system satisfy the following system of equations:

(5) $$\begin{array}{c} bm\left(1-I\right)-\beta S^{p}I-bS+q^{\prime }\delta I=0,\\ \beta e^{-b\tau }S^{p}I+q\delta I-\delta I-\gamma I-\frac{d}{T}I=0. \end{array}$$

Obviously, *E*_0_(*m*, 0) is the disease-free equilibrium of (3). In order to analyze the behavior of the system (3) near *E*_0_, we linearize the system about the equilibrium point; let *S*(*t*) = *X*(*t*) + *m*, *I*(*t*) = *Y*(*t*)

(6) $$\begin{array}{c} X^{\prime }\left(t\right)=-bX\left(t\right)-\left(\beta m^{p}+bm-q^{\prime }\delta \right)Y\left(t\right),\\ Y^{\prime }\left(t\right)=-\left(\delta -q\delta +\gamma +\frac{d}{T}\right)Y\left(t\right)+\beta e^{-b\tau }m^{p}Y\left(t-\tau \right). \end{array}$$

*λ*_1_ = −*b* < 0 is one of the eigenvalues of the linearization of system (6) near the steady state *E*_0_, and the other eigenvalue *λ*_2_ is determined by equation:

(7) $$\begin{array}{c} \lambda -\beta m^{p}e^{-(b+\lambda )\tau }+\delta -q\delta +\gamma +\frac{d}{T}=0. \end{array}$$

Let

(8) $$\begin{array}{c} f\left(\lambda \right)=\lambda -\beta m^{p}e^{-(b+\lambda )\tau }+\delta -q\delta +\gamma +\frac{d}{T}; \end{array}$$

if *ℜ*_0_ > 1, it is easy to show that, for *λ* real,

(9) $$\begin{array}{c} f\left(0\right)=\left(\delta -q\delta +\gamma +\frac{d}{T}\right)\left(1-{ℜ}_{0}\right)<0,\\ \underset{\lambda \to +\infty }{\lim}f\left(\lambda \right)=+\infty ; \end{array}$$

hence, *f*(*λ*) = 0 has a positive real root. Therefore, if *ℜ*_0_ > 1, the disease-free equilibrium *E*_0_(*m*, 0) is unstable.

If *ℜ*_0_ < 1, we prove that the disease-free equilibrium *E*_0_(*m*, 0) is locally stable. Otherwise, *Reλ* ≥ 0. We note that

(10) Reλ=(δ−qδ+γ+dT)(ℜ0e−Reλτcos⁡(lmλτ)−1)≤(δ−qδ+γ+dT)(ℜ0−1),

a contradiction. Hence, the disease-free equilibrium *E*_0_ is locally asymptotically stable if *ℜ*_0_ < 1.

**Theorem 2**

If *ℜ*_0_ < 1, the disease-free equilibrium *E*_0_(*m*, 0) of system (3) is globally asymptotically stable for all *τ* ≥ 0.

To proof the global stability of the disease-free equilibrium *E*_0_(*m*, 0), we choose Lyapunov function

(11) $$\begin{array}{c} V\left(t\right)=X\left(t\right)+Y\left(t\right)+\beta e^{-b\tau }m^{p}\int_{t-\tau }^{t}Y\left(\xi \right)d\xi , \end{array}$$

and it is easy to prove  *V*′(*t*) < 0, lim⁡_*t*→*∞*_ *V*(*t*) = 0; it follows that lim⁡_*t*→*∞*_ *X*(*t*) = 0, lim⁡_*t*→*∞*_ *Y*(*t*) = 0.

### 2.2. Endemic Equilibrium and Its Stability

If *ℜ*_0_ > 1, then system (3) has a unique positive equilibrium *E*_1_(S*, I*), where

(12) S∗=((δ−qδ+γ+(d/T))ebτβ)1/p,I∗=b(bm−q′δ)+(δ−qδ+γ+(d/T))ebτ  ×(m−((δ−qδ+γ+(d/T))ebτβ)1/p).

**Theorem 3**

If *ℜ*_0_ > 1, conditions (17) and (22) are satisfied, then for *τ* ≥ 0 the endemic equilibrium *E*_1_(S*, I*) of system (3) is locally asymptotically stable.

**Proof**

Let *S*(*t*) = *X*(*t*) + S*, *I*(*t*) = *Y*(*t*) + I*; the linearized system is obtained

(13) X′(t)=−(b+βpI∗S∗(p−1))X(t) −(βS∗p+bm−q′δ)Y(t),Y′(t)=βpe−bτI∗S∗(p−1)X(t−τ)+βe−bτS∗pY(t−τ)−(δ−qδ+γ+dT)Y(t).

From the linearized system we obtain the characteristic equation

(14) $$\begin{array}{c} \lambda^{2}+p\lambda +r+\left(g\lambda +q\right)e^{-\lambda \tau }=0, \end{array}$$

where

(15) p=δ−qδ+γ+dT+βpS∗(p−1)I∗+b,r=(b+βpI∗S∗(p−1))(δ−qδ+γ+dT),g=−βS∗pe−bτ,q=−(b+βpI∗S∗(p−1))βe−bτS∗p+(βS∗p+bm−q′δ)βpe−bτI∗S∗(p−1).

For *τ* = 0 the characteristic equation becomes

(16) $$\begin{array}{c} \lambda^{2}+\left(p+g\right)\lambda +\left(r+q\right)=0, \end{array}$$

and we can see that both roots are negative and real if and only if

(17) $$\begin{array}{c} p+g>0,\;\;r+q>0. \end{array}$$

Now for *τ* ≠ 0, if *λ* = *ωi* is a root of (16), we have

(18) $$\begin{array}{c} -\omega^{2}+qe^{-\omega \tau i}+p\omega i+r+g\omega e^{-\omega \tau i}=0. \end{array}$$

Separating the real and imaginary parts, we have

(19) $$\begin{array}{c} r-\omega^{2}+g\omega \sin\left(\omega \tau \right)+q\cos\omega \tau =0,\\ p\omega +g\omega \cos\omega \tau -q\sin\omega \tau =0. \end{array}$$

Adding both equations and regrouping by powers of *ω*, we obtain the following fourth degree polynomial

(20) $$\begin{array}{c} \omega^{4}+\left(p^{2}-g^{2}-2r\right)\omega^{2}+r^{2}-q^{2}=0, \end{array}$$

from which we have

(21) $$\begin{array}{c} \omega^{2}=\frac{g^{2}-p^{2}+2r\pm \sqrt{{\left(g^{2}-p^{2}+2r\right)}^{2}-4\left(r^{2}-q^{2}\right)}}{2}. \end{array}$$

It follows that if

(22) $$\begin{array}{c} p^{2}-g^{2}-2r>0,\;\;r^{2}-q^{2}>0, \end{array}$$

are satisfied, (20) does not have positive solutions, and the characteristic equation (14) does not have purely imaginary roots. Inequalities in (17) and (22) guarantee that all roots of (14) have no positive roots. According to Rouche's theorem, Theorem 3 is proved.

Subsequently we discuss the sufficient conditions under which the endemic equilibrium is globally asymptotically stable for the system (3). For *S* + *E* + *I* + *R* = 1, hence, the dynamics of system (3) in the first octant of *R*_4_ is equivalent to that of the following system:

(23) $$\begin{array}{c} S^{\prime }\left(t\right)=bm\left(1-I\right)-\beta S^{p}I-bS+q^{\prime }\delta I,\\ E^{\prime }\left(t\right)=\beta S^{p}I-\beta e^{-b\tau }S^{p}\left(t-\tau \right)I\left(t-\tau \right)-bE,\\ I^{\prime }\left(t\right)=\beta e^{-b\tau }S^{p}\left(t-\tau \right)I\left(t-\tau \right)+q\delta I-\delta I-\gamma I-\frac{d}{T}I. \end{array}$$

The initial conditions for system (23) take the form

(24) $$\begin{array}{c} S\left(\xi \right)=\varphi_{1}\left(\xi \right),\;\;E\left(\xi \right)=\varphi_{2}\left(\xi \right),\;\;I\left(\xi \right)=\varphi_{3}\left(\xi \right),\\ \varphi_{1}\left(0\right)>0,\;\varphi_{2}\left(0\right)>0,\;\varphi_{3}\left(0\right)>0, \end{array}$$

where (*φ*_1_(*ξ*), *φ*_2_(*ξ*), *φ*_3_(*ξ*)) ∈ *C*([−*τ*, 0], *R*_+0_^3^), the space of continuous functions mapping the interval [−*τ*, 0] into *R*_+0_^3^, where *R*_+0_^3^ = {(*x*_1_, *x*_2_, *x*_3_) : *x*_*i*_ ≥ 0, *i* = 1,2, 3}.

For continuity of the initial conditions, we require

(25) $$\begin{array}{c} E\left(0\right)=\int_{-\tau }^{0}\beta e^{b\xi }S{\left(\xi \right)}^{p}I\left(\xi \right)d\xi . \end{array}$$

It is well known by the fundamental theory of functional differential equations [26], the system (23) has a unique solution (*S*(*t*), *E*(*t*), *I*(*t*)) satisfying the initial conditions. It is easy to show that all solutions of system (23) with initial conditions are defined on [0, +*∞*) and remain positive for all *t* ≥ 0.

**Lemma 4 (see [25])**

Let the initial condition be *S*(*ξ*) = *S*(0) > 0, *E*(*ξ*) = *E*(0) > 0 and *I*(0) > 0, for all *ξ* ∈ [−*τ*, 0). Then *S*(*t*) ≤ max⁡{1, *S*(0) + *E*(0) + *I*(0)} = *M*.

**Theorem 5**

Let the initial condition be *S*(*ξ*) = *S*(0) > 0, *E*(*ξ*) = *E*(0) > 0 and *I*(0) > 0, for all *ξ* ∈ [−*τ*, 0). Further suppose *ℜ*_0_ > 1; then for any infectious period *τ* satisfying

(26) τ>max⁡{1bln⁡βMp−1(ρM+2pI∗+3ρpI∗)βpI∗Mp−1−4bρ−ρ(bm−q′δ),    1bln⁡βMp−1(ρ+1)(I∗p+M)4ρb+2b−βpI∗Mp−1,   1bln⁡βMp−1(I∗p+2Mp+3M)2(δ−qδ+γ+dT)+ρ(bm−q′δ)},

where *M* = max⁡{1, *S*(0) + *E*(0) + *I*(0)}, the endemic equilibrium is globally asymptotically stable.

**Proof**

Let *S*(*t*) = *X*(*t*) + S*, *E*(*t*) = *Y*(*t*) + E*, *I*(*t*) = *Z*(*t*) + I*; the linearized system is:

(27) X′(t)=−(b+βpI∗S∗(p−1))X(t)+(q′δ−βS∗p−bm)Z(t),Y′(t)=βpI∗S∗(p−1)X(t)+βS∗pZ(t)−βpe−bτI∗S∗(p−1)X(t−τ)−βe−bτS∗pZ(t−τ)−bY(t),Z′(t)=βpe−bτI∗S∗(p−1)X(t−τ)+βe−bτS∗pZ(t−τ)−(δ−qδ+γ+dT)Z(t).

The trivial solution of system (27) is globally asymptotically stable and is equivalent to the fact that the endemic equilibrium (S*, E*, I*) of system (23) is globally asymptotically stable. We will employ Lyapunov functional technique to prove it.

Now let us introduce the following functions:

(28) V1(t)=12ρ(X(t)+Y(t))2+12(Y2(t)+Z2(t)),V2(t)=(ρ+1)βpe−bτI∗M(p−1)∫t−τtX2(ξ)dξ+(ρ+1)βe−bτMp∫t−τtZ2(ξ)dξ,

where *ρ* > 0 is an arbitrary real constant. Choosing *ρ* = *βpS* ^∗*p*^/(*bm* − *q*′*δ*), the derivative of *V*_1_(*t*) is

(29) V1′(t)=ρ(X(t)+Y(t))(X′(t)+Y′(t)) +Y(t)Y′(t)+Z(t)Z′(t)=ρ(X(t)+Y(t)) ×[−bX(t)+(q′δ−bm)Z(t)−bY(t)−βpe−bτI∗S∗(p−1)X(t−τ)−βe−bτS∗pZ(t−τ)] +Y(t)[βpI∗S∗(p−1)X(t)+βS∗pZ(t)−βpe−bτI∗S∗(p−1)X(t−τ)−βe−bτS∗pZ(t−τ)−bY(t)] +Z(t)[βpe−bτI∗S∗(p−1)X(t−τ)+βe−bτS∗pZ(t−τ)−(δ−qδ+γ+dT)Z(t)]=−bρX2(t)−(bρ+b)Y2(t)−[δ−qδ+γ+dT]Z2(t)  +[βpI∗S∗(p−1)−2ρb]X(t)Y(t)+ρ(q′δ−bm)Z(t)X(t)−ρβpe−bτI∗S∗(p−1)X(t)X(t−τ)−βρe−bτS∗pX(t)Z(t−τ)−βρe−bτS∗pY(t)Z(t−τ)−ρβpe−bτI∗S∗(p−1)Y(t)X(t−τ)−βpe−bτI∗S∗(p−1)Y(t)X(t−τ)−βe−bτS∗pY(t)Z(t−τ)+βpe−bτI∗S∗(p−1)X(t−τ)Z(t)+βe−bτS∗pZ(t−τ)Z(t),

and applying Cauchy-Chwartz inequality to all product terms, we obtain the following expression:

(30) V1′(t)≤−[2bρ−12βpI∗S∗(p−1)+12ρ(bm−q′δ)    −12βρe−bτMp−1(I∗p+M)]X2(t)−[2ρb+b−12pβI∗Mp−1  −12(ρ+1)βe−bτMp−1(I∗p+M)]Y2(t)−[δ−qδ+γ+dT+12ρ(bm−q′δ)  −12βe−bτMp−1(I∗p+M)]Z2(t)+(ρ+1)βpe−bτI∗M(p−1)X2(t−τ)+(ρ+1)βe−bτMpZ2(t−τ).

We choose Lyapunov function to be the form *V*(*t*) = *V*_1_(*t*) + *V*_2_(*t*), and we get

(31) V′(t)=V1′(t) +(ρ+1)βpe−bτI∗M(p−1)×(X2(t)−X2(t−τ))+(ρ+1)βe−bτMp×(Z2(t)−Z2(t−τ)).

Substituting this in the inequality for *V*_1_(*t*), we get

(32) V′(t)≤−[2bρ+12ρ(bm−q′δ)−12βpI∗M∗(p−1)  −12βe−bτMp−1(ρM+2pI∗+3ρpI∗)]X2(t)−[2ρb+b−12pβI∗Mp−1  −12(ρ+1)βe−bτMp−1(I∗p+M)]Y2(t)−[δ−qδ+γ+dT+12ρ(bm−q′δ)  −12βe−bτMp−1(I∗p+2Mρ+3M)]Z2(t).

The right-hand expression of the above inequality is always negative provided that (26) holds. A direct application of the Lyapunov-LaSalle type theorem shows that lim⁡_*t*→*∞*_ *X*(*t*) → 0, lim⁡_*t*→*∞*_ *Y*(*t*) → 0, lim⁡_*t*→*∞*_ *Z*(*t*) → 0. The proof is complete.

## 3. Continuous Treatment and Pulse Vaccination Strategies 

When continuous treatment and pulse vaccination strategies are included in the SEIR epidemic model with the nonlinear infectious force and vertical transmission, it can be written as follows:

(33) $$\begin{array}{c} S^{\prime }\left(t\right)=bm\left(S+R+E\right)-\beta S^{p}I-bS+q^{\prime }\delta I,\\ E^{\prime }\left(t\right)=\beta S^{p}I-\beta e^{-b\tau }S^{p}\left(t-\tau \right)I\left(t-\tau \right)-bE,\\ I^{\prime }\left(t\right)=\beta e^{-b\tau }S^{p}\left(t-\tau \right)I\left(t-\tau \right)+q\delta I-\delta I-\gamma I-\frac{d}{T}I,\\ R^{\prime }\left(t\right)=\gamma I-bR+bm^{\prime }\left(S+R+E\right)+\frac{d}{T}I.\\ \;\;\;\;\;\;\;\;\;\;\;\;\;\;\;\;t\neq kT,\;k\in Z^{+},\\ S\left(t^{+}\right)=\left(1-\theta \right)S\left(t\right),\;\;E\left(t^{+}\right)=E\left(t\right),\\ I\left(t^{+}\right)=I\left(t\right),\;\;R\left(t^{+}\right)=R\left(t\right)+\theta S\left(t\right),\\ \;\;\;\;\;\;\;\;\;\;\;t=kT,\;k\in Z^{+}, \end{array}$$

and *S*(*t*), *E*(*t*), *I*(*t*), and *R*(*t*) are the number of susceptible, exposed, infectious, and recovered at time *t*, respectively. *θ* is the proportion of those vaccinated successfully at *kT*, which is called pulse vaccination rate.

We also consider the following reduced systems:

(34) $$\begin{array}{c} S^{\prime }\left(t\right)=bm\left(1-I\right)-\beta S^{p}I-bS+q^{\prime }\delta I,\\ I^{\prime }\left(t\right)=\beta e^{-b\tau }S^{p}\left(t-\tau \right)I\left(t-\tau \right)+q\delta I-\delta I-\gamma I-\frac{d}{T}I,\\ \;\;\;\;\;\;\;\;\;\;\;\;\;\;\;\;t\neq kT,\;k\in Z^{+},\\ S\left(t^{+}\right)=\left(1-\theta \right)S\left(t\right),\;\;I\left(t^{+}\right)=I\left(t\right),\\ \;\;\;\;\;\;\;\;\;t=kT,\;k\in Z^{+}. \end{array}$$

Let *Ω* be the following subset of *R*_+_^2^, *Ω* = {(*S*, *I*) ∈ *R*_+_^2^ | *S* ≥ 0, *I* ≥ 0, *S* + *I* ≤ 1}. From biological considerations, we discuss system (34) in the closed set *Ω*. It can be verified that *Ω* is positively invariant with respect to system (34).

We first state two important lemmas which are useful in our following discussions.

**Lemma 6 (see [12])**

Consider the following impulsive differential equation

(35) $$\begin{array}{c} u^{\prime }\left(t\right)=a-bu\left(t\right),\;t\neq kT,\\ u\left(t^{+}\right)=\left(1-\theta \right)u\left(t\right),\;t=kT, \end{array}$$

where *a* > 0, *b* > 0, 0 < *θ* < 1. Then the above system has a unique positive periodic solution given by

(36) $$\begin{array}{c} u^{*}\left(t\right)=\frac{a}{b}+\left(\bar{u}-\frac{a}{b}\right)e^{-b(t-kT)},\;kT<t\leq \left(k+1\right)T, \end{array}$$

which is globally asymptotically stable, where

(37) $$\begin{array}{c} \bar{u}=\frac{a}{b}\frac{\left(1-\theta \right)\left(1-e^{-bT}\right)}{1-\left(1-\theta \right)e^{-bT}}. \end{array}$$

**Lemma 7 (see [27, 28])**

Consider the following equation:

(38) $$\begin{array}{c} u^{\prime }\left(t\right)=a_{1}u\left(t-\tau \right)-a_{2}u\left(t\right), \end{array}$$

where *a*_1_, *a*_2_, *τ* > 0, *u*(*t*) > 0 for −*τ* ≤ *t* ≤ 0. One has

1. if *a*_1_ < *a*_2_, then lim⁡_*t*→*∞*_ *u*(*t*) = 0;
2. if *a*_1_ > *a*_2_, then lim⁡_*t*→*∞*_ *u*(*t*) = +*∞*.

### 3.1. Global Stability of the Disease-Free Periodic Solution

Now we will prove the disease-free periodic solution (*S*(*t*), 0) is global attractively. We first demonstrate the existence of the disease-free periodic solution, in which infectious individuals are entirely absent from the population permanently, that is, *I*(*t*) ≡ 0 for all *t* > 0. Under this condition, the growth of susceptible individuals must satisfy

(39) $$\begin{array}{c} S^{\prime }\left(t\right)=bm-bS,\;t\neq kT,\;\;k\in Z^{+},\\ S\left(t^{+}\right)=\left(1-\theta \right)S\left(t\right),\;t=kT,\;\;k\in Z^{+}. \end{array}$$

By Lemma 6, we obtain the periodic solution of system (39),

(40) $$\begin{array}{c} S^{*}\left(t\right)=m-\frac{m\theta e^{-b(t-nT)}}{1-\left(1-\theta \right)e^{-bT}},\;kT<t\leq \left(k+1\right)T, \end{array}$$

and this solution is globally asymptotically stable. Hence, the system (34) has a disease-free periodic solution (S*(*t*), 0).

**Theorem 8**

Let (*S*(*t*), *I*(*t*)) be any solution of (34); then the disease-free periodic solution (S*(*t*), 0) is globally asymptotically stable provided that

(41) $$\begin{array}{c} {ℜ}^{*}=\frac{m^{p}e^{-b\tau }\beta }{\delta -q\delta +\gamma +\left(d/T\right)}{\left(\frac{1-e^{-bT}}{1-\left(1-\theta \right)e^{-bT}}\right)}^{p}<1. \end{array}$$

**Proof**

Since ℜ* < 1, we can choose *ɛ*_0_ > 0 sufficiently small such that

(42) $$\begin{array}{c} e^{-b\tau }\beta {\left(\frac{m\left(1-e^{-bT}\right)}{1-\left(1-\theta \right)e^{-bT}}+{ɛ}_{0}\right)}^{p}<\delta -q\delta +\gamma +\frac{d}{T}. \end{array}$$

From the first equation of system (34), we have *S*′(*t*) < *bm* − *bS*, and then we consider the following comparison system with pulse:

(43) $$\begin{array}{c} w^{\prime }\left(t\right)=bm-bw\left(t\right),\;t\neq kT,\\ w\left(t^{+}\right)=\left(1-\theta \right)w\left(t\right),\;t=kT,\\ w\left(0^{+}\right)=S\left(0^{+}\right). \end{array}$$

In view of Lemma 6, we obtain *S*(*t*) ≤ *w*(*t*) and

(44) lim⁡t→∞w(t)=m−mθe−b(t−nT)1−(1−θ)e−bT=S∗(t),          kT<t≤(k+1)T.

There exists an integer *k*_1_, such that

(45) $$\begin{array}{c} S\left(t\right)\leq w\left(t\right)<S^{*}\left(t\right)+{ɛ}_{0}. \end{array}$$

That is,

(46) S(t)<S∗(t)+ɛ0≤m(1−e−bT)1−(1−θ)e−bT+ɛ0:=S¯,      kT<t≤(k+1)T, k>k1.

Furthermore, from the second equation, we have

(47) I′(t)≤βe−bτS¯pI(t−τ)−(δ−qδ+γ+dT)I(t),t≠kT, k>k1,

and then we consider the following comparison equation:

(48) w1′(t)=βe−bτS¯pI(t−τ)−(δ−qδ+γ+dT)I(t),t≠kT, k>k1;

then, from (41) we have $\beta e^{-b\tau }{\bar{S}}^{p}<\delta -p\delta +\gamma +(d/T)$. In view of Lemma 7, we have *w*_1_′(*t*) < 0, lim⁡_*k*→*∞*_ *I*(*t*) = 0. So there must exist an integer *k*_2_ > *k*_1_, such that *I*(*t*) < *ɛ*_1_ for all *t* > *k*_2_ *T*.

When *t* > *k*_2_ *T*, from the first equation of system (34), we have

(49) $$\begin{array}{c} S^{\prime }\left(t\right)>\left(bm-\left(bm-q^{\prime }\delta \right){ɛ}_{1}\right)-\left(\beta {\bar{S}}^{p-1}{ɛ}_{1}+b\right)S\left(t\right). \end{array}$$

Consider the following comparison impulsive differential equation for all *t* > *k*_2_ *T*

(50) w2′(t)=(bm−(bm−q′δ)ɛ1)−(βS¯p−1ɛ1+b)w2(t), t≠kT,w2(t+)=(1−θ)w2(t), t=kT,w2(0+)=S(0+).

By Lemma 6, we have the unique periodic solution of system (50) given by

(51) w2∗=bm−(bm−q′δ)ɛ1βS¯p−1ɛ1+b(1−θe−(ɛ1βS¯p−1+b)(t−nT)1−(1−θ)e−(ɛ1βS¯p−1+b)T),kT<t≤(k+1)T. 

By the comparison theorem, there exists an integer *k*_3_ > *k*_2_ such that

(52) $$\begin{array}{c} S\left(t\right)>w_{2}\left(t\right)>w_{2}^{*}-{ɛ}_{1},\;kT<t\leq \left(k+1\right)T. \end{array}$$

Because *ɛ*_0_ and *ɛ*_1_ are sufficiently small, it follows from (46) and (52) that lim⁡_*t*→*∞*_ *S*(*t*) = S*(*t*). Therefore, the disease-free solution (S*(*t*), 0) of system (34) is globally attractive. The proof is completed.

Denote

(53) θ∗=(ebT−1)((βmpebτ(δ−qδ+γ+(d/T)))1/p−1),τ∗=1bln⁡βmpebτ(δ−qδ+γ+(d/T))(1−e−bT1−(1−θ)e−bT)p.

**Corollary 9**

(i) If *βm* ^*p*^ ≤ *e* ^*bτ*^(*δ* − *qδ* + *γ* + (*d*/*T*)), then the infection-free periodic solution is globally attractive.

(ii) If *βm* ^*p*^ > *e* ^*bτ*^(*δ* − *qδ* + *γ* + (*d*/*T*)), then the infection-free periodic solution is globally attractive provided that *θ* > θ*.

**Corollary 10**

(i) If *βm* ^*p*^((1 − *e* ^−*bT*^)/(1 − (1 − *θ*)*e* ^−*bT*^))^*p*^ ≤ *e* ^*bτ*^(*δ* − *qδ* + *γ* + (*d*/*T*)), then the infection-free periodic solution is globally attractive.

(ii) If *βm* ^*p*^((1 − *e* ^−*bT*^)/(1 − (1 − *θ*)*e* ^−*bT*^))^*p*^ > *e* ^*bτ*^(*δ* − *qδ* + *γ* + (*d*/*T*)), then the disease will be endemic and system (34) is permanent provided that *τ* > τ*.

Theorem 8 determines the global attractivity of (34) in *Ω* for the case ℜ* < 1. Its epidemiological implication is that the infectious population vanishes in time so the disease dies out. Corollaries 9 and 10 imply that the disease will disappear if the vaccination rate or the length of latent period of the disease is large enough.

### 3.2. Persistent

In this section we say the disease is endemic if the infectious population persists above a certain positive level for sufficiently large time. The endemicity of the disease can be well captured and studied through the notion of uniform persistence.

**Definition 11**

System (34) is said to be uniformly persistent if there exist positive constants *M*_*i*_ ≥ *m*_*i*_, *i* = 1,2, (both are independent of the initial values), such that every solution (*S*(*t*), *I*(*t*)) with positive initial conditions of system (34) satisfies

(54) $$\begin{array}{c} m_{1}\leq S\left(t\right)\leq M_{1},\;\;m_{2}\leq I\left(t\right)\leq M_{2}. \end{array}$$

Denote

(55) $$\begin{array}{c} {ℜ}_{*}=\frac{\beta }{e^{b\tau }\left(\delta -q\delta +\gamma +\left(d/T\right)\right)}{\left(\frac{\left(1-\theta \right)\left(1-e^{-bT}\right)}{1-\left(1-\theta \right)e^{-bT}}\right)}^{p}. \end{array}$$

**Theorem 12**

If *ℜ*_∗_ > 1, then there is a positive constant *m*_*I*_ such that each positive solution (*S*(*t*), *I*(*t*)) of system (34) satisfies *I*(*t*) ≥ *m*_*I*_ for all t sufficiently large.

**Proof**

Let (*S*(*t*), *I*(*t*)) be any solution with initial values of system (34), and then it is obvious that *S*(*t*) ≤ 1, *I*(*t*) ≤ 1 for all *t* > 0. We are left to prove there exist positive constants *m*_*S*_, *m*_*I*_ and *t*_0_ (*t*_0_ is sufficiently large) such that *S*(*t*) ≥ *m*_*S*_, *I*(*t*) ≥ *m*_*I*_ for all *t* > *t*_0_.

Firstly, from the first equation of system (34), we have

(56) $$\begin{array}{c} S^{\prime }\left(t\right)>q^{\prime }\delta -\left(\beta +b\right)S. \end{array}$$

Consider the following comparison equations:

(57) $$\begin{array}{c} X^{\prime }\left(t\right)=q^{\prime }\delta -\left(\beta +b\right)X\left(t\right),\;t\neq kT,\\ X\left(t^{+}\right)=\left(1-\theta \right)X\left(t\right),\;t=kT. \end{array}$$

By Lemma 6 and the comparison theorem [29], we know that for any sufficiently small *ɛ* > 0, there exists a *t*_0_ (*t*_0_ is sufficiently large) such that

(58) S(t)≥X(t)>X∗(t)−ɛ≥q′δβ+b((1−θ)(1−e−(β+b)T)1−(1−θ)e−(β+b)T)−ɛ=mS>0.

Now, we will prove that there exist *m*_*I*_ > 0 and a sufficiently large *t*_0_ such that *I*(*t*) ≥ *m*_*I*_ for all *t* > *t*_0_. Since the proof is rather long, it will be convenient to divide it into two steps.

*Step  1.* Since *ℜ*_∗_ > 1, there exist *m*_I_* > 0, $\bar{ɛ}>0$ sufficiently small such that

(59) $$\begin{array}{c} \beta \eta^{p}e^{-b\tau }-\left(\delta +\gamma +\frac{d}{T}-q\delta \right)>0, \end{array}$$

where $\eta =(q^{\prime }\delta /\left(\beta m_{I}^{*}\;+\;b\right))(\left(1\;-\;\theta )(1\;-\;e^{-bT}\right)/\left(1\;-\;(1\;-\;\theta )e^{-bT}\right))\;-\bar{ɛ}$.

We claim that for any *t*_0_ > 0, it is impossible that *I*(*t*) < *m*_I_* for all *t* ≥ *t*_0_. Suppose that the claim is not valid. There exists a *t*_0_ > 0 such that *I*(*t*) < *m*_I_* for all *t* ≥ *t*_0_, and then follows from the first equation of system (34) that for *t* ≥ *t*_0_,

(60) $$\begin{array}{c} S^{\prime }\left(t\right)>q^{\prime }\delta -\left(\beta m_{I}^{*}+b\right)S\left(t\right). \end{array}$$

Consider the comparison impulsive system for *t* ≥ *t*_0_,

(61) $$\begin{array}{c} X_{1}^{\prime }\left(t\right)=q^{\prime }\delta -\left(\beta m_{I}^{*}+b\right)X\left(t\right),\;t\neq kT,\\ X_{1}\left(t^{+}\right)=\left(1-\theta \right)X_{1}\left(t\right),\;t=kT. \end{array}$$

According to Lemma 6, there exists *T*_1_ > *t*_0_ such that

(62) S(t)>X1∗(t)−ɛ¯≥q′δβmI∗+b((1−θ)(1−e−β(mI∗+b)T)1−(1−θ)e−β(mI∗+b)T)−ɛ¯=η,

for all *T*_1_ > *t*_0_. The second equation of system (34) can be translated into the following form:

(63) I′(t)=(βSp(t)e−bτ−δ+qδ−γ−dT)I(t)−βe−bτddt∫t−τtSp(ξ)I(ξ)dξ.

Define a function *V*(*t*) such that

(64) $$\begin{array}{c} V\left(t\right)=I\left(t\right)+\beta e^{-b\tau }\int_{t-\tau }^{t}S^{p}\left(\xi \right)I\left(\xi \right)d\xi ; \end{array}$$

then the derivative of *V*(*t*) along the solution of system (34) is

(65) V′(t)=(βSp(t)e−bτ−δ+qδ−γ−dT)I(t)=(δ−qδ+γ+dT)(βSp(t)e−bτδ−qδ+γ+(d/T)−1)I(t),t>T1.

From (55), we obtain *V*′(*t*) > 0, *t* > *T*_1_, which implies that *V*(*t*) → *∞*, *t* → *∞*. This is contrary to *V*(*t*) < 1 + *βτe* ^−*bτ*^. Hence, there exists a *t*_1_ > 0 such that *I*(*t*_1_) ≥ *m*_I_*.

*Step  2.* According to Step 1, for any positive solution (*S*(*t*), *I*(*t*)) of system (34), we are left to consider two cases. First, if *I*(*t*) > *m*_I_* for all *t* > *t*_1_, then our aim is obtained. Second *I*(*t*) oscillates about *m*_I_* for all large *t*. In this case, setting t* = inf⁡_*t*>*t*_1__ *I*(*t*) ≤ *m*_I_*, there are two possible cases for t*.

Define

(66) $$\begin{array}{c} m_{I}=\min\left\{\frac{m_{I}^{*}}{2},q_{1}\right\},\;q_{1}=m_{I}^{*}e^{-(\delta -q\delta +\gamma +(d/T))\tau }. \end{array}$$

We hope to show that *I*(*t*) ≥ *m*_*I*_ for all large *t*. The conclusion is evident in the first case. For the second case, let t* > 0 and *ρ* > 0 satisfy *I*(t*) = *I*(t* + *ρ*) = *m*_I_*, and *I*(*t*) < I*, *S*(*t*) > *η* for t* < *t* < t* + *ρ*. Therefore, it is certain that there exists a *g* (0 < *g* < *τ*) such that

(67) $$\begin{array}{c} I\left(t\right)\geq \frac{m_{I}^{*}}{2}\;\text{for}\;\;t^{*}<t<t^{*}+g. \end{array}$$

In this case, we will discuss three possible cases in terms of the sizes of *g*, *ρ*, and *τ*.

*Case  1.* If *ρ* ≤ *g* < *τ*, then *I*(*t*) ≥ (*m*_I_*/2) for t* < *t* < t* + *ρ*.

*Case  2.* If *g* ≤ *ρ* ≤ *τ*, then from the second equation of system (34), we can deduce *I*′(*t*)>−(*δ* − *qδ* + *γ* + (*d*/*T*))*I*(*t*) for *t* ∈ [t*, t* + *τ*] and *I*(t*) = *m*_I_*, and it is obvious that *I*(*t*) ≥ *q*_1_ for t* < *t* < t* + *g*.

*Case  3.* If *g* ≤ *T* ≤ *ρ*, we will consider the following two cases, respectively.

*Subcase  3.1.* For t* < *t* < t* + *τ*, it is easy to obtain *I*(*t*) > *q*_1_.

*Subcase  3.2.* For t* + *τ* < *t* < t* + *ρ*, it is easy to obtain *I*(*t*) > *q*_1_. Then, proceeding exactly as the proof for the above claim, we see that *I*(*t*) ≥ *m*_*I*_ for t* + *τ* < *t* < t* + *ρ*. Since this kind of interval [t*, t* + *ρ*] is chosen in an arbitrary way (we only need t* to be large), we conclude that *I*(*t*) ≥ *m*_*I*_ for all large *t* in the second case. In view of our above discussions, the choices of *m*_*I*_ are independent of the positive solution, and we have proved that any positive solution of (34) satisfies *I*(*t*) ≥ *m*_*I*_ for all large *t*. The proof is completed.

Set

(68)   θ∗=(ebT−1)(1−((ebτ(δ−qδ+γ+(d/T)))/β)1/p)((ebτ(δ−qδ+γ+(d/T)))/β)1/p+(ebT−1),τ∗=1bln⁡βebτ(δ−qδ+γ+(d/T))((1−θ)(1−e−bT)1−(1−θ)e−bT)p.

From Theorems 12, we also easily obtain the following results.

**Corollary 13**

(i) If *βe* ^−*bτ*^ > *δ* − *qδ* + *γ* + (*d*/*T*), then the disease will be endemic and system (34) is permanent provided that *θ* < *θ*_∗_.

(ii) If *β*((1 − *θ*)(1 − *e* ^−*bT*^)/(1 − (1 − *θ*)*e* ^−*bT*^))^*p*^ > *δ* − *qδ* + *γ* + (*d*/*T*), then the disease will be endemic and system (34) is permanent provided that *τ* < *τ*_∗_.

## 4. Numerical Simulations

In this section, we present some numerical simulations to demonstrate our theoretical results established in this paper. 

**Figure 1:**
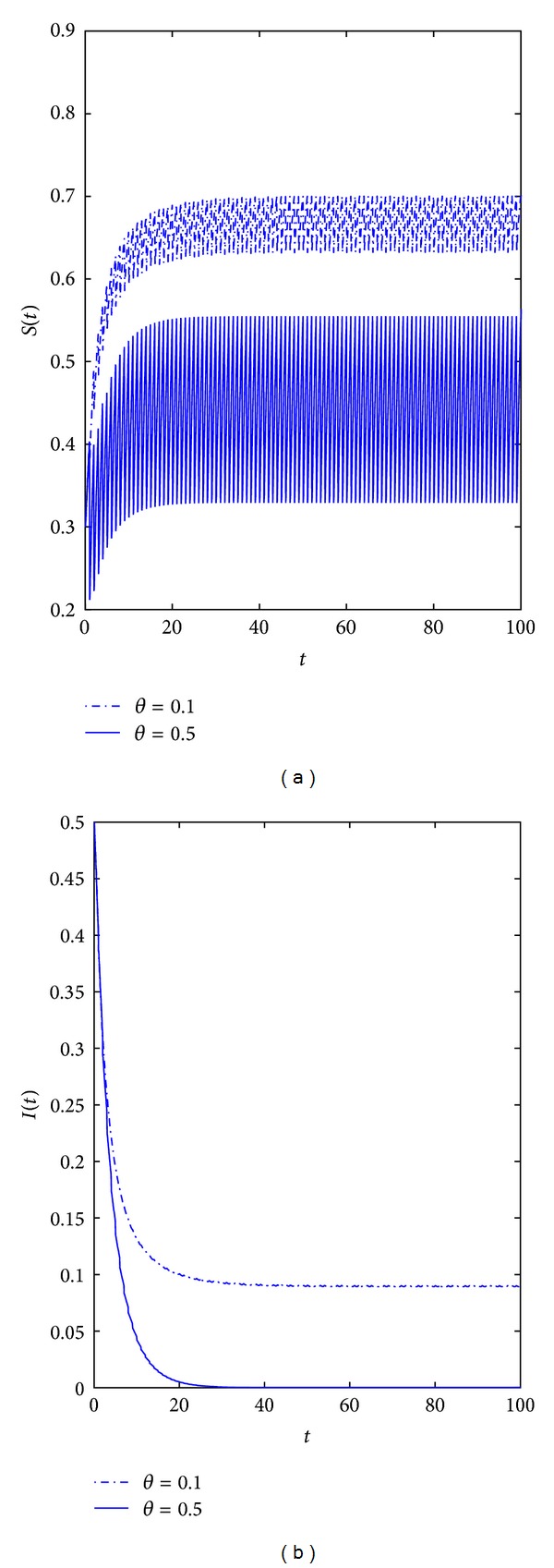
Dynamical behavior of the system (34) with *b* = 0.5, *m* = 0.85, *m*′ = 0.15, *τ* = 0.5, *β* = 0.7, *q*′ = 0.5, *q* = 0.5, *γ* = 0.05, *δ* = 0.1, *T* = 2, and *p* = 3. (a) Time-series of the susceptible population with *θ* = 0.1,  0.5 respectively. (b) Time-series of the infective population with *θ* = 0.1,  0.5.

*Example  1*. Letting *b* = 0.5, *m* = 0.85, *m*′ = 0.15, *τ* = 0.5, *β* = 0.7, *q*′ = 0.5, *q* = 0.5, *γ* = 0.05, *δ* = 0.1, *T* = 2, *p* = 3, we consider the pulse vaccination strategy (see Figure 1), the result shows that the disease fades away when the proportion of those vaccinated successfully *θ* = 0.5; but the disease will exist everlasting when the proportion of those vaccinated successfully *θ* = 0.1. So, this verifies the results in Corollaries 9 and 13, for the epidemic disease with vertical transition periodical vaccination is an effective method to prevent the disease. Also, it can be seen that with the increase of *θ* which typically causes oscillation bigger of the susceptible. 

**Figure 2:**
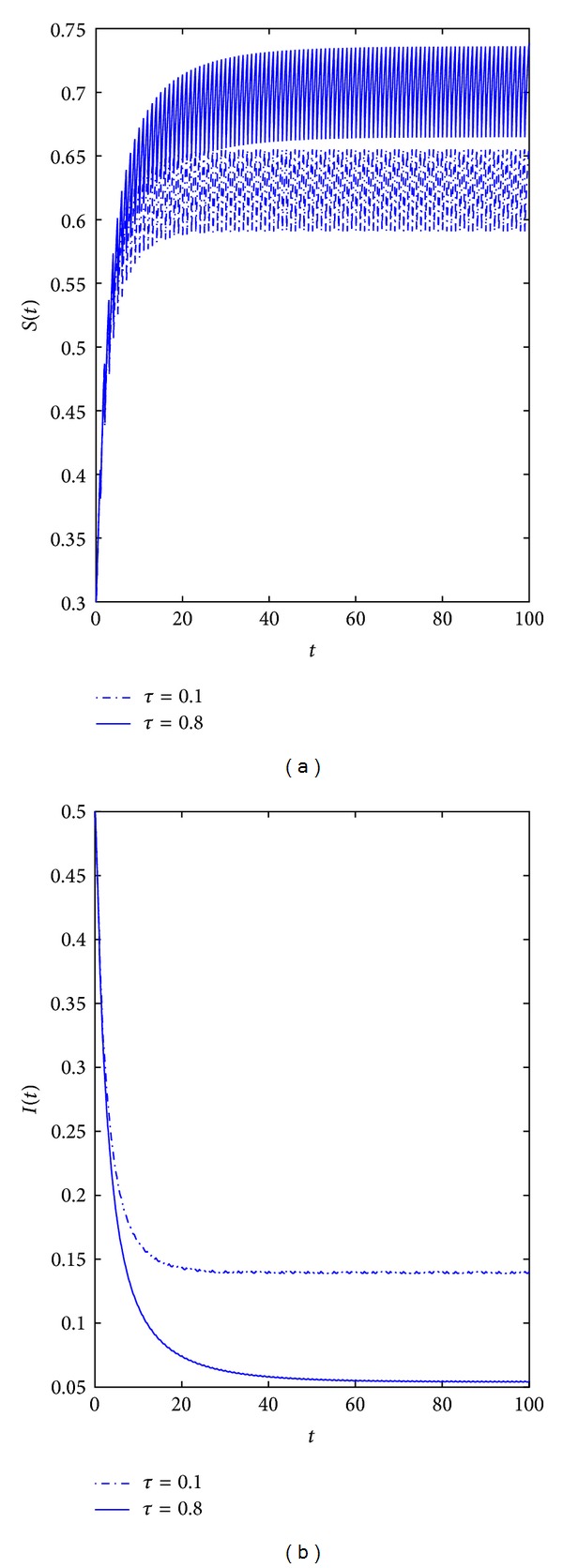
Dynamical behavior of the system (34) with *b* = 0.5, *m* = 0.85, *m*′ = 0.15, *θ* = 0.1, *β* = 0.7, *q*′ = 0.5, *q* = 0.5, *γ* = 0.05, *δ* = 0.1, *T* = 2, and *p* = 3. (a) Time-series of the susceptible population with *τ* = 0.1,0.8, respectively. (b) Time-series of the infective population with *τ* = 0.1,  0.8.

*Example  2*. We use the same parameters as in Example  1 except choosing *θ* = 0.1 and *τ* = 0.1,0.8, respectively. Compare *τ* = 0.1 with *τ* = 0.8 is obviously that the longer of the latent period the lower of the infective number (see Figure 2), and this shows disease with long latent period disadvantage to the spread of the disease. 

**Figure 3:**
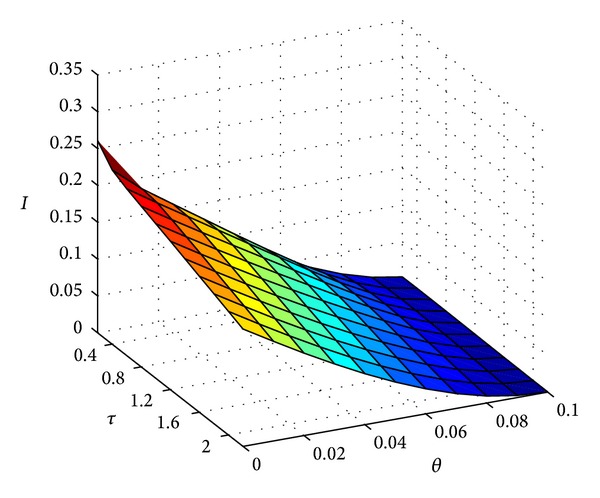
The value of infectious individual with *t* = 100, *b* = 0.5, *m* = 0.85, *m*′ = 0.15, *β* = 0.7, *q*′ = 0.5, *q* = 0.5, *γ* = 0.05, *δ* = 0.1, *T* = 2, *p* = 3, *θ* ∈ [0,0.1], *τ* ∈ [0,0.8].

*Example  3*. For the same parameters as in Example  1 and take *θ* ∈ [0,0.1] and *τ* ∈ [0,0.8], we obtain the number of infected individual (see Figure 3), when *t* = 100. With the increase of *θ* and *τ* the number of infected individual is decreasing, so these results indicate that it will be helpful to control the disease with vertical transition for bigger *θ* and *τ*.

## 5. Conclusion

In this paper, for a class of epidemic disease with latent period and vertical transition, we present two delayed SEIR epidemic models with nonlinear incidence rate based on the spread characters of the disease (such as tuberculosis). Our model is more approach to the realistic problem which is different from [17, 19]. Moreover, the methods in our model are different from the existing results because more factors are considered. When only considering constant treatment, we obtain basic reproductive number *ℜ*_0_ and prove the global stability by using the Lyapunov functional method. For the SEIR model with pulse vaccination we also get the theoretical result, if ℜ* < 1, the disease-free periodic solution is globally attractive; and if *ℜ*_∗_ > 1, the disease is permanent by using the comparison theorem of impulsive differential equation. By some simulation experiments, it clearly shows that the larger of the proportion of those vaccinated successfully the lower of the infective individuals, and the longer of the latent period the lower of the infective individuals. So these results demonstrate that it will be helpful to control the disease with vertical transition for bigger *θ* and *τ*.
